# Growth and Body Composition of Artificially-Reared Lambs Exposed to Three Different Rearing Regimens

**DOI:** 10.3390/ani11123370

**Published:** 2021-11-24

**Authors:** Hitihamy M. G. P. Herath, Sarah J. Pain, Paul R. Kenyon, Hugh T. Blair, Patrick C. H. Morel

**Affiliations:** 1 School of Agriculture and Environment, Massey University, Private Bag 11222, Palmerston North 4442, New Zealand; S.J.Pain@massey.ac.nz (S.J.P.); P.R.Kenyon@massey.ac.nz (P.R.K.); H.Blair@massey.ac.nz (H.T.B.); P.C.Morel@massey.ac.nz (P.C.H.M.); 2 Department of Livestock Production, Faculty of Agricultural Sciences, Sabaragamuwa University of Sri Lanka, Belihuloya 70140, Sri Lanka

**Keywords:** carcass and viscera composition, commercial milk replacer, cost analysis, early weaning, fat deposition, neutral detergent fibre, pellet fibre level, stomach components

## Abstract

**Simple Summary:**

Optimum lamb growth and cost-effective artificial lamb rearing are important to maximise farm profit. In addition to milk feeding, supplementation of a solid feed with balanced nutrients and an adequate level of fibre will aid rumen development, allowing for a smooth transition from a liquid to a solely solid diet. This study aimed to determine the effect of pellet fibre level, milk replacer composition and early milk weaning at 42 days of age, on the growth and body composition of lambs reared artificially to 57 days of age. Results demonstrate that lamb growth rate to 57 days of age was not affected by early weaning or pellet fibre level. Early milk weaning increased pellet intake and empty rumen weight. Early weaning resulted in leaner carcasses due to reduced fat deposition and lower total feed costs compared to lambs offered milk to 57 days of age. Overall, feeding lambs either low or high fibre pellets and weaning early (42 days of age) was shown to be beneficial, as growth was similar to unweaned lambs, but leaner carcasses and reduced feed costs were observed.

**Abstract:**

This study was designed to investigate the influence of pellet fibre level, milk replacer composition and age at weaning on growth and body composition of lambs reared artificially. Romney ram lambs were randomly allocated to one of three rearing treatments; HFP57: commercial milk replacer to 57 days of age, and high fibre concentrate pellets; HFP42: commercial milk replacer with early weaning at 42 days of age, and high fibre concentrate pellets; LFP42: high protein milk replacer from 2–16 days of age followed by commercial milk replacer with early weaning at 42 days of age, and low fibre concentrate pellets. Lambs were slaughtered at 57 days of age. Overall average daily liveweight gain of lambs did not differ (*p* > 0.05) between treatments. Dressing out percentage, carcass weight, empty small intestine and omental fat were higher (*p* < 0.05) in HFP57 than in both HFP42 and LFP42 lambs. HFP42 and LFP42 lambs had heavier (*p* < 0.05) empty rumen weights. Whole body protein content was higher (*p* < 0.05) in HFP42 lambs compared to both HFP57 and LFP42 lambs. Fat content and daily fat deposition were greater (*p* < 0.05) in HFP57 lambs than HFP42 and LFP42 lambs. Weaning lambs at 42 days of age with provision of either low or high fibre concentrate pellets, resulted in similar growth rates, reduced whole body fat deposition and was a more cost-effective rearing regimen.

## 1. Introduction

Artificial rearing of lambs is necessary in cases of orphaned and mismothered lambs and is becoming an increasing requirement in the sheep dairy industry [1,2]. Traditionally, fulfilment of a lamb’s nutrient requirement was considered to depend on milk intake due to negligible solid feed intake in the first few weeks of life [3]. Consequently, many studies have focused on optimisation of milk composition to improve lamb growth [4,5]. However, lambs have higher crude protein to metabolisable energy ratio (CP:ME) requirement during their first few weeks of life than current typical commercial lamb milk replacers, with static CP:ME, provide [4]. When CP:ME requirements are either met or exceeded, improved lamb growth rates are observed [5]. Moreover, lambs fed commercial milk replacers could not compensate for the imbalance of CP:ME during their first two weeks of age through solid feed, as little or no intake occurs and the rumen is not yet sufficiently developed. However, pellet intake has been reported to reach considerable levels (approximately 200 g/d/lamb) by 30–40 days of age in lambs fed a diet of milk replacer and pellets [6].

Early solid feed intake improves lamb growth and rumen development [7,8,9,10]. This prepares lambs for a smoother transition to their post-weaning diet [11] and minimises potential post-weaning growth check due to better nutrient utilization through a more developed rumen and increased fermentative capacity. Thus, if pellet intake is substantial, it may be possible to wean lambs from milk replacer earlier without compromising their growth. Early milk weaning of artificially reared lambs would benefit farmers by shortening the milk feeding phase, reducing milk replacer costs and the labour costs associated with milk feeding. Previous studies have focused on artificial rearing regimens for calves to a greater extent than lambs [12]. Optimal and cost-effective weaning and nutritional regimens (milk and solid feed) for artificially reared lambs require further investigation to ensure not only optimum growth and development pre-weaning occurs, but also appropriate post-weaning growth.

Roughage supplementation is reported to improve growth and rumen development in young ruminants [13,14]. Although roughage inclusion dilutes the energy density of lambs’ rations, which could impair early-life growth. Thus, provision of low levels of fibre in a concentrate diet could be a strategy to improve growth performance pre- and post-weaning, whilst also ensuring adequate rumen development. Porter et al. [15] reported that digestibility of a calf diet was higher when they were fed a low fibre starter compared to high fibre starter. Further, Castells et al. [16] also showed that average daily liveweight gains of pre-weaned calves tended to be greater when fed a low fibre pelleted starter diet, compared to calves fed a high fibre starter diet. However, there is limited research on pellet fibre level and growth performance of lambs. Xie et al. [17] reported that lambs grew at a similar rate from 21 to 60 days of age when they were provided a pelleted diet containing either 14 or 18% neutral detergent fibre (NDF) in combination with milk replacer feeding. Increasing the NDF level of the pellets to 22 or 26% increased the starter intake of lambs and resulted in greater average daily gain (ADG) during the same period compared to lambs fed the low NDF pellets (14% NDF). Pellet NDF level did not have any effect on feed efficiency of lambs in the same study from days 21 to 60 of age.

The objective of this study was investigate the effect of three different artificially rearing regimens (HFP57: commercial milk replacer to 57 days of age, and high fibre concentrate pellets; HFP42: commercial milk replacer with early weaning at 42 days of age, and high fibre concentrate pellets; LFP42: high protein milk replacer from 2–16 days of age followed by commercial milk replacer with early weaning at 42 days of age, and low fibre concentrate pellets) on the growth and body composition of lambs. Additionally, the cost effectiveness of each rearing regimen was determined.

## 2. Materials and Methods

The experiment was carried out at Massey University, Palmerston North, New Zealand from August 2019 to October 2019. The research procedures used were approved by the Massey University Animal Ethics Committee (MUAEC 19/64).

### 2.1. Animal Management

The experiment encompassed three different phases: pre-weaning, milk weaning, and post-weaning as shown in Table 1.

#### 2.1.1. Pre-Weaning

Twenty-seven Romney ram lambs born to twin-bearing ewes were selected for the study. Lambs were allowed to suckle from their dam for the first 24 h after birth before one of the lambs’ in the twin set was separated from its dam to enter the study. The 27 individual lambs were collected from their dams over a four-day period (28 August 2019 to 31 August 2019).

The selected lambs (mean live weight (LW) 4.93 ± 0.22 kg) were moved in-doors, individually penned and randomly allocated to one of three rearing treatments (Table 1); (i) HFP57 (*n* = 9): commercial milk replacer, high fibre concentrate pellets to 57 days of age; (ii) HFP42 (*n* = 9): commercial milk replacer, high fibre concentrate pellets and early weaning from the milk replacer at 42 days of age; (iii) LFP42 (*n* = 9): high protein milk replacer from 2–16 days of age followed by commercial milk replacer, low fibre pellets and early weaning from milk replacer at 42 days of age. All lambs were fed milk replacer at 2.1 times their maintenance energy requirement based on their LW as per previous studies by our group [5,6]. The maintenance requirement was calculated as ME_m_ = 0.40 MJ/kgLW^0.75^d^−1^ [18]. The commercial milk replacer contained 969.8 g/kg dry matter, 262.9 g/kg crude protein (CP), 275 g/kg fat, 56.2 g/kg ash, 350.4 g/kg lactose, 11.4 g/kg calcium, 6.1 g/kg phosphorous, 22.7 MJ/kg gross energy, 21.8 MJ/kg metabolisable energy (ME) and 12.05 g/MJ CP:ME on fresh matter basis (Milligans Feed Ltd., Oamaru, New Zealand). The high protein milk replacer was an 80:20 blend of the commercial milk replacer powder with a powdered milk protein concentrate (Fonterra, Auckland, New Zealand). The high protein milk replacer contained 967.4 g/kg dry matter, 324.1 g/kg CP, 20.84 MJ/kg ME (CP:ME 15.55 g/MJ), 215.4 g/kg fat, 61.2 g/kg ash, 343.6 g/kg lactose, 13.6 g/kg calcium, 7.2 g/kg phosphorous, and 21.7 MJ/kg gross energy on fresh matter basis. Metabolisable energy content was calculated as metabolisability coefficient multiplied by gross energy considering metabolisability coefficient of milk replacer and milk protein concentrate as 0.96 [4]. To prepare the milk, the powdered milk replacer was mixed with warm tap water at a ratio of 1/4 (*w*/*w*) [4,5,18]. Lambs were bottle-fed five times daily (at 8.00 a.m., 11.00 a.m., 2.30 p.m., 6.30 p.m., and 9.00 p.m.) up to two weeks of age, then four times daily (at 8.00 a.m., 11.00 a.m., 2.30 p.m., and 6.00 p.m.) up to the milk-weaning phase (days 38 to 42) for HFP42 and LFP42 lambs and until the end of experiment for HFP57 lambs.

Lambs were provided *ad libitum* access to either high fibre concentrate pellets (HFP57 and HFP42 lambs), or low fibre concentrate pellets (for LFP42 lambs) from four days of age to slaughter at approximately 57 days of age. The high fibre concentrate pellet was a blend of broll, soya bean, molasses, barley, and limestone (acid detergent fibre (ADF) 69 g/kg, NDF 209 g/kg; Denver Stock Feeds, Palmerston North, Table 2). The low fibre concentrate pellet was a blend of skim milk powder, soya bean, molasses, barley and limestone (ADF 44 g/kg, NDF 117 g/kg; Denver Stock Feeds, Palmerston North, Table 2). The lambs had free access to water at all times.

Individual milk, pellet and lucerne chaff intakes were recorded daily. Lamb LW was recorded twice weekly.

#### 2.1.2. Milk Weaning (Days 38 to 42) and Post-Weaning (Days 43 to 57) Phases for HFP42 and LFP42 Treatments

Milk weaning (38 to 42 days of age) and post-weaning (43 to 57 days of age) phases were applicable only for HFP42 and LFP42 treatments (Table 1), while HFP57 lambs continued to be provided with their full milk allowance until slaughter at 57 days of age. During the milk-weaning phase, lambs in HFP42 and LFP42 treatments were offered 50% of their milk allowance for five days from day 37. Lambs in HFP42 and LFP42 treatments were bottle-fed twice daily at 8.00 a.m. and 6.00 p.m. and fully weaned from milk at 42 days. All lambs were provided with *ad libitum* access to either high (HFP57 and HFP42 lambs) or low fibre concentrate pellets (LFP42 lambs) during both the milk weaning and post weaning phases. Lucerne chaff (115 g/kg CP and 7.14 MJ/kg ME; Oaklane Stables Premium Chaff, Hawkes Bay, New Zealand, Table 2) was offered (40 g/d) for lambs from 38 to 57 days of age. All lambs were reared until approximately 57 days of age.

Samples of commercial milk replacer (*n* = 4) and concentrate pellets (*n* = 5 high fibre and *n* = 3 low fibre concentrate pellets) were collected by sub-sampling each milk replacer/pellet bag at unpacking. Three composite samples of milk replacer, high fibre, and low fibre concentrate pellets were prepared by pooling all the samples collected during the experiment. Three samples from high protein milk replacer and lucerne chaff were collected. All collected feed samples were stored at −20 °C until analysis for proximate composition.

#### 2.1.3. Faecal Sample Collection

Fresh faecal samples were collected from each lamb at slaughter and were stored at −20 °C until they were analysed for dry matter, gross energy, and acid insoluble ash contents. Details on chemical analysis methods are included in the analysis of sample section.

### 2.2. Slaughter

Three lambs out of 27 were excluded from the experiment due to health reasons (*n* = 1 and *n* = 2 from HFP57 and LFP42 treatments, respectively). All lambs were weighed and their crown to rump length, rib length, and abdominal girth measured, after being fasted for 12 h.

The lambs were slaughtered via captive bolt, exsanguinated, skinned, and eviscerated. Weights of the head, feet, and tail (combined), whole skin, hot carcass, total viscera, blood, and full stomach were recorded. Weights of the liver, kidneys, heart, lungs, and testis, spleen, and omental fat were also measured for each individual.

**Figure 1:**
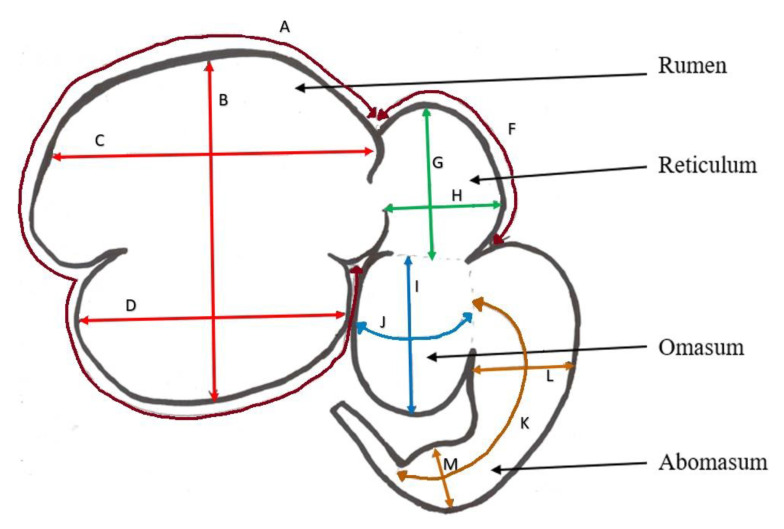
Length, width and circumference measurements of rumen, reticulum, omasum, and abomasum recorded at slaughter (A; circumference of rumen, B; length of rumen, C; width of dorsal rumen, D; width of ventral rumen, F; circumference of reticulum, G; length of reticulum, H; width of reticulum, I; length of omasum, J; circumference of omasum, K; length of abomasum, L; width at proximal end of abomasum, M; width at distal end of abomasum, red, green, blue and brown lines represent measurements recorded for rumen, reticulum, omasum and abomasum, respectively and dark brown lines represent circumference of rumen and reticulum, respectively).

Length, width, circumference and height measurements of the rumen, reticulum, omasum, and abomasum were recorded (Figure 1) after placing the whole full stomach on a flat surface before separating each stomach component. Individual weights of the full and empty rumen, reticulum, omasum, reticulum, small intestine, and large intestine were recorded.

Carcasses were cut in half longitudinally in the middle of the spine and the weights of each half recorded. A half from each carcass, total viscera, blood, head, feet, and whole skin and skin samples were stored at −20 °C in sealed plastic bags until being further processed. Prior to processing, the frozen carcass and viscera plus blood were cut into small blocks by a band saw and ground separately through a 3 mm grinding plate (Hobart Manufacturing Company, Troy, OH, USA). Subsamples of the ground carcass and viscera plus blood were collected and stored at −20 °C until analysis for proximate composition. The head, feet, tail, and skin samples were not analysed.

### 2.3. Analysis of Samples

Feed, body tissue and faecal samples were analysed at the Nutrition Laboratory, Massey University, Palmerston North, New Zealand.

#### 2.3.1. Feed Samples

Commercial milk replacer, high protein milk replacer, low and high fibre concentrate pellets and lucerne chaff samples were analysed for dry matter content by drying the sample at 105 °C in an oven (methods 930.15 and 925.10, [19]) and ash content in a furnace at 550 °C (method 942.05, [19]), crude protein content using the Dumas method (method 968.06, [19]) and gross energy content by bomb calorimetry.

Fat content of commercial milk replacer and high protein milk replacer were determined by the Mojonnier extraction method (method 922.06, [19]) and fat content of concentrate pellets was determined by the Soxtec extraction method (method 2003.06, [19]). Lactose content of commercial milk replacer and high protein milk replacer was determined using an enzymatic method (Boehringer Mannheim/R-Biofarm Enzyme kit for Lactose/D-Galactose—enzymatic digestion colorimetric was determined at 340 nm). Calcium and phosphorous content of commercial milk replacer and high protein milk replacer were determined by colorimetric method (sample preparation according to method 968.08D, [19]). The mineral content (calcium, magnesium, potassium, sodium, phosphorus, sulphur and chloride) of pellets was determined by inductively coupled plasma emission spectrometry (Thermo iCAP 6000 series ICP-OES, Thermo Fisher Scientific, Waltham, MA, USA). The NDF, ADF, and lignin content of pellets and lucerne chaff were determined using the Fibretec System (method 2002.04 and 973.18, [19]).

Acid insoluble ash content of feed samples were analysed using the method described by Sullivan and Carpenter [20]. Briefly, ashed samples were digested in acid, filtered through an ash-less filter paper and re-ashed, and the acid insoluble ash determined. The acid insoluble ash was used as an indigestible marker [21].

#### 2.3.2. Body Tissue Samples

Carcass and viscera plus blood samples were analysed for dry matter content by drying the sample at 105 °C in an oven (methods 950.46B, [19]) and ash content in a furnace at 550 °C (method 920.153 and 923.03, [19]), crude protein content by the Dumas method (method 968.06, [19]), fat content by the Soxtec extraction method (method 991.36, [19]) and gross energy content by bomb calorimetry.

#### 2.3.3. Faecal Samples

Faecal samples of five lambs, which had the highest pellet intakes from each treatment were freeze dried (Cuddon FB18, Cuddon Freeze dry, McArteny street, Blenheim, New Zealand). The freeze-dried samples were ground by Variable Speed Rotor Mill (Fritsch Pulverisette 14, Fritsch GmbH, Idar-Oberstein, Germany) at 16,000 rpm and sieved through a 0.5 mm sieve. Those samples were analysed for dry matter content and gross energy content as reported for feed samples.

Acid insoluble ash content of faecal samples were analysed using the method described by Sullivan and Carpenter [20]. The acid insoluble ash was used as an indigestible marker [21].

### 2.4. Calculations

Individual milk replacer, pellet and lucerne chaff intakes during the experiment were calculated as the daily amount offered minus refusal. Metabolisable energy content of pellets and lucerne chaff were calculated as gross energy multiplied by their metabolisability coefficient, considering metabolisability coefficient 0.68 for pellet [4], 0.459 for lucerne chaff [22]. Daily ME and CP intakes were calculated based on milk replacer, pellet and lucerne chaff intake multiplied by their respective ME and CP concentrations. The cumulative ME and CP intakes were calculated by summation of daily ME and CP intake from the different feed types. Daily NDF and ADF intakes were calculated based on pellet and lucerne chaff intake multiplied by their respective NDF and ADF concentrations. The cumulative NDF and ADF intakes were calculated by summation of daily NDF and ADF intake from the different feed types.

Energy digestibility of feed was calculated using following equation considering acid insoluble ash as an indigestible marker.

Energy digestibility of feed = {(Gross energy feed/Indicator in feed) − (Gross energy faeces/Indicator in faeces)}/(Gross energy feed/Indicator in feed),(1)

Average daily lamb live weight gain during the overall experimental period and 2–37, 38–42, and 43–57 day periods were calculated as the difference between LW at the first and last day of each period divided by the number of days in each phase. The LW gain per kilogram dry matter intake (DMI) was calculated as LW gain during experiment divided by total DMI.

Dressing percentage of carcass was calculated as carcass weight divided by LW at the slaughter (approximately after 12 h since last feed). The gut fill was determined by weighing the stomach (combined weight of rumen, reticulum, omasum, and abomasum) and intestines (duodenum, jejunum, ileum, caecum, colon, and rectum) before and after removal of their contents. Empty stomach weight was determined by summation of empty rumen, reticulum, omasum, and abomasum weights. Percentage of empty stomach components relative to LW at slaughter were calculated.

The volume of each stomach component was calculated as follows, where B; length of rumen, C; width of dorsal rumen, D; width of ventral rumen, G; length of reticulum, H; width of reticulum, I; length of omasum, J; circumference of omasum, K; length of abomasum, L; width at proximal section of abomasum, M; width at distal section of abomasum (Figure 1).

Rumen volume was calculated assuming dorsal and ventral rumen sections as two ellipsoids, reticulum as an ellipsoid, omasum as a cylinder and abomasum as one-half of a cone.

Rumen volume = ((B/4) × (C/2) × (rumen height/2) × (4/3) × π) + ((B/4) × (D/2) × (rumen height/2) × (4/3) × π),(2)

Reticulum volume = ((G/2) × (H/2) ∗ (reticulum height/2) × (4/3) × π),(3)

Omasum volume = π × (J/(π × 2))^2^ × I, (4)

Abomasum volume = {(L/2)^2^ + ((L/2) × (M/2)) + (M/2)^2^} × π × (K/3), (5)

The total stomach volume was calculated as summation of volumes of rumen, reticulum, omasum, and abomasum.

The body volume was calculated as follows.

Body volume = π × (Girth/2π) × (Girth/2π) × rib cage width along spine,(6)

Dry matter, water, fat, protein and ash content of carcass and viscera plus blood were calculated by multiplying the weights of carcass and viscera plus blood by their respective analysed chemical composition percentage value. Whole carcass composition was calculated as the summation of compositions of carcass and viscera plus blood. Dry matter, water, fat, protein, and ash content of carcass and viscera plus blood of lambs at the start of study were calculated as initial live weight times the average percentage chemical composition at the start of the trial using baseline data from [5] which were the same genotype lambs and slaughtered after 24 h from birth [5]. The daily deposition of dry matter, water, fat, protein, and ash during the growth of each lamb was calculated as the difference between the amount of dry matter, water, fat, protein, and ash in the body at slaughter and the calculated composition of each lamb at the start of the study, divided by number of days to slaughter [4,5].

The cost of commercial milk replacer or high protein milk replacer, pellets and lucerne chaff for the experimental period were calculated as total intake multiplied by their respective cost per kilogram (commercial milk replacer, high protein milk replacer, high fibre pellets, low fibre pellets and lucerne chaff were 5.52, 6.41, 0.75, 0.85, and 2.06 NZ$/kg, respectively). The total feed cost for the experimental period was calculated by summation of cost for milk replacer or high protein milk replacer, pellets and lucerne chaff. Feed costs per kg liveweight gain was calculated as total feed cost divided by total liveweight gain.

### 2.5. Statistical Analysis

Growth performance, feed and nutrient intakes data collected throughout the experiment were used to calculate average daily live weight gain, or feed and nutrient intake values as mentioned in 2.4 calculation section and those daily average values were analysed using a linear model with treatment as a fixed effect (Proc GLM, SAS 9.4, Cary, NC, USA [23]), considering individual lamb as the experiment unit. Slaughter parameters, volumes of stomach components, daily nutrient deposition in carcass and viscera plus blood samples and feed cost were analysed using a linear model with treatment as a fixed effect (Proc GLM, SAS 9.4, Cary, NC, USA [23]), considering individual lamb as the experiment unit. Carcass composition was analysed with a linear model with treatment as a fixed effect and carcass weight as a covariate by Proc GLM, SAS 9.4, Cary, NC, USA [23]. Viscera plus blood composition was analysed with a linear model with treatment as a fixed effect considering viscera plus blood weight as a covariate by Proc GLM, SAS 9.4, Cary, NC, USA [23]. Differences were identified considering confidence level as 0.05, where appropriate, using the least significant difference (LSD) mean comparison test.

## 3. Results

### 3.1. Intake and Growth Performance

#### 3.1.1. Pre-Weaning Phase (2–37 Days)

The dry matter intake (DMI) from either milk replacer or pellets, daily ME, CP, ADF, and NDF intakes and DMI from lucerne chaff and total DMI did not differ between treatments at 2 to 37 days of age (Table 3). The combined CP:ME intake was higher (*p* < 0.05) in LFP42 lambs compared to both HFP57 and HFP42 lambs, which did not differ. The initial live weight, ADG and LW gain per kilogram DMI did not differ between treatments.

#### 3.1.2. Early Weaning Phase (38–42 Days)

The DMI from milk replacer was higher (*p* < 0.05) in HFP57 lambs than both HFP42 and LFP42 lambs because HFP42 and LFP42 lambs were being weaned off milk during this period (Table 3). The lambs in HFP42 had higher (*p* < 0.05) DMI from pellets than HFP57 lambs while LFP42 lambs did not differ from either HFP57 or HFP42 lambs. The ME intake of lambs did not differ between treatments. The CP intake of HFP57 lambs was greater (*p* < 0.05) than HFP42 and LFP42 lambs, which did not differ. The combined CP:ME intake did not differ between treatments.

The HFP42 lambs had higher (*p* < 0.05) daily ADF and NDF intakes compared to HFP57 and LFP42 lambs, which did not differ. The DMI from lucerne chaff and total DMI did not differ between treatments. The ADG of lambs did not differ between treatments.

#### 3.1.3. Post-Early Weaning Phase (43–57 Days)

During this phase, the diet combination of commercial milk replacer, high fibre concentrate pellets, and lucerne chaff fed to HFP57 lambs had a higher digestibility (0.94), which was significantly different (*p* < 0.05) compared to the combinations of high fibre concentrate pellets and lucerne chaff fed to HFP42 lambs (0.78) and low fibre concentrate pellet, lucerne chaff fed to LFP42 lamb (0.81), which did not differ (Table 3).

The lambs in HFP42 and LFP42 treatments had higher (*p* < 0.05) DMI from pellets than HFP57 lambs (Table 3). Daily ME and CP intakes of lambs were greater (*p* < 0.05) in HFP57 lambs compared to HFP42 and LFP42 lambs, which did not differ. The combined CP:ME intake was higher (*p* < 0.05) in both HFP42 and LFP42 compared to HFP57 lambs. The HFP42 lambs had higher (*p* < 0.05) daily NDF intake during the same period than HFP57 and LFP42 lambs, which did not differ. Daily ADF intakes of HFP42 and LFP42 lambs were higher (*p* < 0.05) than HFP57 lambs. The DMI from lucerne chaff and total DMI did not differ between treatments.

The ADG of HFP57 lambs was greater (*p* < 0.05) than HFP42 and LFP42 lambs, which did not differ. The HFP57 lambs had higher (*p* < 0.05) LW gain per kilogram DMI compared both HFP42 and LFP42 lambs, which did not differ.

#### 3.1.4. Overall Experimental Period (2–57 Days)

##### Feed Intake

Daily milk replacer intake was highest (*p* < 0.05) in HFP57 lambs compared to both HFP42 and LFP42 lambs, which did not differ (Table 4). Daily pellet intake was higher (*p* < 0.05) in both HFP42 and LFP42 than HFP57 lambs. Total lucerne and dry matter intakes (DMI) did not differ between treatments.

##### Crude Protein (CP) Intake

Daily CP intake from milk replacer was higher (*p* < 0.05) in HFP57 than HFP42 and LFP42 lambs (Table 4). Daily CP intake from pellets was higher (*p* < 0.05) in both early-weaned lamb groups (HFP42 and LFPF) compared to HFP57 lambs. The CP intake from lucerne chaff did not differ between treatments. Daily CP intake from milk replacer, pellet and lucerne chaff did not differ between treatments.

##### Metabolisable (ME) and Digestible Energy Intake

Daily ME intake from milk replacer was higher (*p* < 0.05) in HFP57 compared to HFP42 and LFP42 lambs (Table 4). Daily ME intake from pellet was higher (*p* < 0.05) in both early-weaned groups (HFP42 and LFP42) than HFP57 lambs. Daily ME intake from lucerne chaff did not differ between treatments. Total ME intake was greater (*p* < 0.05) in HFP57 compared to LFP42 lambs and HFP42 lambs did not differ either from HFP57 or LFP42 lambs. The digestible energy intake was higher (*p* < 0.05) in HFP57 lambs compared to HFP42 and LFP42 lambs, which did not differ. The combined total CP:ME intake from milk replacer, pellet and lucerne chaff did not differ between treatments.

##### Acid Detergent Fibre (ADF) and Neutral Detergent Fibre (NDF) Intake

The ADF and NDF intakes from pellets were greater (*p* < 0.05) in HFP42 lambs compared to both HFP57 and LFP42 lambs, which did not differ (Table 4). The ADF and NDF intakes from lucerne chaff did not differ between treatments. Daily NDF intake was greater (*p* < 0.05) in HFP42 lambs compared to both HFP57 and LFP42 lambs, which did not differ.

##### Growth Performance

The LW at slaughter and average daily gain (ADG) did not differ between treatments (Table 4). The LW gain per kilogram DMI was greater (*p* < 0.05) in HFP57 lambs compared to both HFP42 and LFP42 lambs.

### 3.2. Slaughter Parameters

The crown to rump length, abdominal girth, and ribs length did not differ (*p* > 0.05) between treatments (Table 5). Carcass weight, dressing, full and empty weights of small intestine and omental fat were greater (*p* < 0.05) in HFP57 lambs compared to HFP42 and LFP42 lambs, which did not differ. All the other slaughter parameters did not differ between treatments.

Empty rumen weight was higher (*p* < 0.05) in HFP42 and LFP42 lambs compared to HFP57 lambs (Table 6). Full omasum was heavier (*p* < 0.05) in HFP42 lambs compared to HFP57 lambs. The LFP42 lambs did not differ either from HFP57 or HFP42 lambs.The weights of rumen, reticulum, and omasum relative to live weight of lambs at slaughter were greater (*p* < 0.05) in both HFP42 and LFP42 lambs compared to HFP57 lambs. The abomasum weight relative to the live weight at slaughter was higher (*p* < 0.05) in both HFP57 and HFP42 lambs compared to LFP42 lambs. Lambs in HFP42 and LFP42 treatments had heavier (*p* < 0.05) rumens relative to their total empty stomach weight, compared to HFP57 lambs. The HFP57 lambs had a heavier (*p* < 0.05) abomasum relative to the total empty stomach weight compared to both HFP42 and LFP42 lambs, which did not differ (*p* > 0.05). The calculated reticulum volume tended to be higher (*p* = 0.077) in HFP42 and LFP42 lambs compared to HFP57 lambs. The abomasum volume tended to be higher (*p* = 0.063) in HFP57 and HFP42 lambs compared to LFP42 lambs. All the other stomach parameters did not differ (*p* > 0.05) between treatments (Table 6).

### 3.3. Body Composition

#### 3.3.1. Nutrient Content

##### Carcass

Protein content of the carcass was greater (*p* < 0.05) in HFP42 lambs compared to both HFP57 and LFP42 lambs, which did not differ (Table 7). Fat content of the carcass was greater (*p* < 0.05) in HFP57 lambs compared to both HFP42 and LFP42 lambs, which did not differ. Dry matter, ash, water, and gross energy contents of carcass did not differ between treatments.

##### Viscera plus Blood

Fat and gross energy contents of viscera plus blood were higher (*p* < 0.05) in HFP57 lambs compared to both HFP42 and LFP42 lambs, which did not differ (Table 7). Dry matter, protein, ash, and water contents of viscera plus blood did not differ between treatments.

##### Whole Body (Carcass and Viscera plus Blood)

Protein content of whole body was higher (*p* < 0.05) in HFP42 lambs compared to HFP57 and LFP42 lambs, which did not differ (Table 7). Fat content of whole body was greater (*p* < 0.05) in HFP57 lambs compared to both HFP42 and LFP42 lambs, which did not differ. Dry matter, ash, water, and gross energy contents of whole body did not differ between treatments.

#### 3.3.2. Nutrient Deposition Rate

Daily fat deposition in the carcass, viscera plus blood and whole body were greater (*p* < 0.05) in HFP57 lambs compared to both HFP42 and LFP42 lambs, which did not differ (Table 8). Daily dry matter, protein, ash, and water depositions in carcass, viscera plus blood and whole body did not differ between treatments.

### 3.4. Cost Analysis

The cost of milk replacer per lamb and total cost of feed per lamb were highest (*p* < 0.05) in HFP57 treatment compared to both HFP42 and LFP42 treatments (Table 9). The cost of pellets was higher (*p* < 0.05) in both HFP42 and LFP42 lambs compared to HFP57 lambs. The cost of lucerne chaff did not differ between treatments.

## 4. Discussion

### 4.1. Intake and Growth Performance

#### 4.1.1. Pre-Weaning Phase (2–37 Days of Age)

There was no difference in ADG between treatments up until the early weaning of HFP42 and LFP42 lambs. During this period, all the lambs had access to commercial milk replacer/high protein milk replacer and low or high fibre concentrate pellets. There was no difference between treatments for either milk replacer or pellet intake. This explains the lack of difference in ADG and live weight (LW) gain per kilogram DMI.

Higher CP:ME intake early in a lamb’s life has been reported to improve its growth rate [4,5]. During the first two weeks of the study, LFP42 lambs were fed a high protein milk replacer (CP:ME 15.55 g/MJ), with their CP intake from milk being higher. When milk intake and pellet intake were combined, LFP42 lambs had a higher CP:ME intake compared to both HFP57 and HFP42 lambs. However, the higher CP:ME intake of LFP42 lambs during first two weeks did not result in improved growth rate compared to HFP57 and HFP42 lambs. This suggests that lambs may require a higher CP:ME intake for a longer period (63 and 57 days, for [5] and [6] studies, respectively) to have a notable growth difference. Alternatively, the commercial milk replacer (CP:ME ratio 12.2 MJ ME/kg) used in this study provided an adequate CP:ME for lamb growth, resulting in the lack of difference.

#### 4.1.2. Early Weaning Phase (38–42 Days of Age)

The HFP42 and LFP42 lambs were weaned from milk over a period of five days (38–42 days of age). During this period, ADG between early weaned and un-weaned lambs did not differ. Early weaned lambs were offered only half an allowance of 2.1 times of their maintenance energy requirement via the commercial milk replacer and so to meet their nutrient requirements for growth, they increased their pellet intake. The boosted pellet intake compensated the nutrient requirements of HFP42 and LFP42 lambs, leading to similar ADG between treatments.

#### 4.1.3. Post-Early Weaning Phase (43–57 Days of Age)

The ADG of HFP42 and LFP42 lambs was lower than HFP57 over this period. The HFP57 lambs were still being offered commercial milk replacer during this period while HFP42 and LFP42 lambs were not. Daily pellet DMI of HFP42 and LFP42 lambs was more than twice that of HFP57 lambs; however, there was no difference between treatments for total daily DMI. The HFP57 lambs had greater combined CP and ME intake from milk replacer, pellets and lucerne chaff than the early weaned lambs. This led to higher ADG and feed efficiency for growth in HFP57 lambs compared to both HFP42 and LFP42 lambs during this period, because of the higher nitrogen (N) and energy utilization efficiency of milk than pellet diets, as found by Danso et al. [16] which reported 73 vs. 30%, and 96 vs. 71% utilization efficiency for N and energy, respectively.

Pellet DMI at 42 days of age relative to LW at the start of the experiment was 2.2, 5.5, and 3.9%, respectively for HFP57, HFP42, and LFP42 lambs. Greenwood, et al. [24] reported no significant post-weaning growth check in calves if they reached a pellet intake of 1.0–2.0% of their birth weight, at weaning. Although HFP42 and LFP42 lambs in the present study had higher pellet intakes than the range suggested by [24], a post-early weaning growth check was observed. Pellet compositions (crude protein, ash, and fat content) of the present study and that of [24] were approximately similar. Unfortunately, [24] did not report the energy content and digestibility of the pellet used in their study, making it difficult to explain the growth check observed in lambs in the present study, despite their higher pellet intake. It may simply be there is a difference between calves and lambs.

#### 4.1.4. Overall Experimental Period (2–57 Days of Age)

Lambs weaned at 42 days of age and fed pellets containing either low or high fibre had similar overall ADG and LW at slaughter (57 days) as their counterparts fed milk replacer and high fibre pellets. Early weaned lambs (HFP42 and LFP42) had consumed approximately half the quantity of milk replacer consumed by HFP57 lambs. The pellet intake of HFP42 and LFP42 lambs over this period was 83 and 64%, respectively, higher than that of HFP57 lambs, resulting in similar total DMI between the treatments. This resulted in similar ADG in the three treatment groups. The higher LW gain per kilogram DMI in HFP57 lambs was due to the higher digestibility of their diet and higher ME intake compared to both HFP42 and LFP42 lambs. This indicates early weaned lambs had lower feed efficiency.

### 4.2. Slaughter Parameters

Carcass weight and dressing out percentage were greater in HFP57 lambs compared to early weaned lambs (i.e., HFP42 and LFP42). This was most likely caused by heavier weights of the rumen, reticulum and omasum relative to LW in both HFP42 and LFP42 lambs compared to HFP57 lambs. Muir, et al. [25] reported a higher dressing out percentage for unweaned lambs compared to weaned lambs and suggested the difference was due to the less rumen development of unweaned lambs, which in consistent with findings of the present study.

Empty small intestine weight was heavier in HFP57 lambs compared to HFP42 and LFP42 lambs in the present study. Attaix and Meslin [26] reported that progressive intestinal maturity of ruminants was observed during the milk feeding phase, not the milk weaning phase. In addition, they observed that the villus was longer in the distal parts of small intestine (jejunum and ileum) of unweaned lambs at eight weeks of age compared to weaned lambs. These results are consistent with the current study which found the small intestine was heavier in lambs fed milk replacer until slaughter compared to those early weaned from milk replacer.

The percentage contribution of the abomasum to the combined stomach weight is higher (60%) at birth and progressively becomes less as a lamb transitions from a milk-based diet to a solid plant-based diet (20%, in adult sheep) due to rumen development and the establishment of fermentation functionality [27]. The HFP42 and LFP42 lambs had lighter abomasal weight (20% vs. 19%, respectively) compared to HFP57 lambs (29%) at slaughter, confirming that early weaning promotes rumen growth and development.

At birth, the reticulo-rumen is about 35% of the whole stomach weight and its contribution increases to 60–65% in adult sheep [27]. In the present study, the percentage weight of rumen relative to whole stomach of HFP42 and LFP42 lambs was approximately 65% while it was 55% for HFP57 lambs. Moreover, HFP42 and LFP42 lambs had heavier rumens relative to their LW at slaughter compared to HFP57 lambs, suggesting that early weaning positively affected their rumen growth and development.

The greater ME intake of HFP57 lambs, and higher digestibility of their diet compared to the diets of HFP42 and LFP42 lambs, resulted in higher omental fat deposition. Gastrointestinal tract (GIT) protein synthesis increases as a lamb’s rumen develops, being 11.5% in a pre-ruminant lamb vs. 18–35% in ruminant lamb [28]. McBride and Kelly [29] suggested this alters the animal’s energy use due to its marked contribution to the animal’s energy cost. Therefore, HFP42 and LFP42 lambs may have had higher energy expenditure than HFP57 lambs, leading to less energy available for omental fat deposition which could reduce the visceral fat content of lambs.

### 4.3. Body Composition

Early weaned lambs had reduced fat content and reduced rates of fat deposition in the carcass, viscera plus blood, and whole body compared to lambs fed milk replacer until slaughter (HFP57). The HFP57 lambs had higher ME and higher digestible energy intake than HFP42 and LFP42 lambs. As mentioned above, HFP42 and LFP42 lambs may have had higher energy expenditure for GIT development and protein synthesis compared to HFP57 lambs. Combined, these differences could have resulted in a lower fat deposition and consequently more lean growth of HFP42 and LFP42 lambs compared to HFP57 lambs.

Protein content of both the carcass and whole body (carcass and viscera plus blood) was higher in HFP42 lambs compared to both HFP57 and LFP42 lambs. The HFP42 lambs would have gained LW mainly by protein deposition rather than fat deposition, which reflects the findings of previous studies on restricted lamb feeding [30,31,32]. Interestingly, the protein content of both the carcass and whole body of the LFP42 lambs was similar to HFP57 lambs, although they were weaned early. Thus, further investigations on effect of pellet fibre level and early weaning on protein content of lamb carcass and whole body are required to clarify the relationships.

Gross energy content of viscera plus blood was reduced in early weaned lambs (HFP42 and LFP42) compared to those fed milk and pellet until slaughter (HFP57). This is due to the higher fat content of viscera plus blood of HFP57 lambs, which contained more energy compared to protein. Although HFP57 carcasses had a higher fat content, the higher protein deposition in HFP42 carcasses increased the energy content, resulting in no difference between treatments for carcass gross energy content.

### 4.4. Cost Analysis

The feed costs of early weaned lambs were lower compared to HFP57 lambs, as early weaned lambs were offered approximately half of the more expensive milk replacer (compared to pellets) quantity of HFP57 lambs. The HFP42 and LFP42 lambs required an additional 4.0 and 4.7 kg/lamb of high and low fibre pellets, respectively, to match the weight of HFP57 lambs. There are no previous studies on lamb feed cost comparisons between early and late-weaned artificial lamb rearing systems. In calf rearing systems, early weaning has been reported to reduce the feed cost compared to late weaned calves [33], which supports the findings of the present study.

## 5. Conclusions

Lambs early weaned at 42 days of age and fed concentrate pellets containing either a low or high fibre level showed similar overall growth to 57 days of age, as that of lambs feed milk replacer and pellets for the whole period. However, the early weaning rearing regimen showed overall reduced feed costs. Additionally, early weaning reduced the fat content and rate of fat deposition in the carcass, viscera plus blood and whole body and was more cost effective than feeding lambs with milk replacer and pellets to 57 days. Thus, early weaning of lambs may benefit the farmers by receiving premium price for leaner carcass at market and reduced feed cost under artificial lamb rearing conditions. However, the effect of early weaning on growth performance of lambs and rumen development subsequent to 57 days of age deserves further study.

## Figures and Tables

**Table 1:** Treatments and different phases of the experiment.

Lambs’ Age (Days)/Treatment	Days 2–37: Pre-Weaning	Days 38–42: Weaning	Days 43–57: Post-Weaning
HFP57	Commercial milk replacer—full allowance, *ad libitum* high fibre concentrate pellets	Commercial milk replacer—full allowance, *ad libitum* high fibre concentrate pellets, Lucerne chaff	Commercial milk replacer—full allowance, *ad libitum* high fibre concentrate pellets, Lucerne chaff
HFP42	Commercial milk replacer—full allowance, *ad libitum* high fibre concentrate pellets	Commercial milk replacer—50% of milk allowance, *ad libitum* high fibre concentrate pellets, Lucerne chaff	No commercial milk replacer, *ad libitum* high fibre concentrate pellets, Lucerne chaff
LFP42	High protein milk replacer—full allowance from day 2 to 15, followed by commercial milk replacer—full allowance only till day 37, *ad libitum* low fibre concentrate pellets	Commercial milk replacer—50% of milk allowance, *ad libitum* low fibre concentrate pellets, Lucerne chaff	No commercial milk replacer, *ad libitum* low fibre concentrate pellets, Lucerne chaff

**Table 2:** Pellet composition and chemical analysis of pellets and lucerne chaff in fresh matter basis.

Ingredient	Low Fibre Concentrate Pellet (LFP)	High Fibre Concentrate Pellet (HFP)	Lucerne Chaff
Pellet composition			
Barley, g/kg	0.270	0.390	-
Broll, g/kg ^1^	0.000	0.351	-
Soya bean meal, g/kg	0.225	0.218	-
Wheat, g/kg	0.389	0.000	-
Molasses, g/kg ^2^	0.030	0.030	-
Skim milk powder, g/kg	0.075	0.000	-
Limestones, g/kg	0.010	0.010	-
Sheep premix, g/kg ^3^	0.001	0.001	-
Chemical composition			
Dry matter, g/kg	891.2	880.5	873.8
Ash, g/kg	60.4	53.5	66.0
Crude protein (CP), g/kg	180.0	185.6	114.7
Fat, g/kg	34.4	47.4	0.00
Minerals, g/kg			
Calcium	6.9	8.6	N/A
Magnesium	3.0	2.0	N/A
Potassium	12.2	10.6	N/A
Sodium	0.3	0.6	N/A
Phosphorus	5.8	4.5	N/A
Sulphur	2.2	2.0	N/A
Chloride, mg/kg	0.16	0.21	N/A
Gross energy, MJ/kg	15. 66	15. 97	15.56
Neutral Detergent Fibre, g/kg	116.8	208.6	543.3
Acid Detergent Fibre, g/kg	44.4	69.3	431.4
Lignin, g/kg	10.2	14.3	92.2
Metabolisable energy (ME), MJ/kg ^4^	10.65	10.86	7.14
CP:ME ratio, g/MJ ^5^	16.91	17.10	16.06

^1^ Broll is a mixture of wheat bran and wheat pollard, crude protein 153 g/kg and neutral detergent fibre 359 g/kg. ^2^ Source of molasses is sugar beet. ^3^ Cobalt 0.2 g/kg, Iodine 0.2 g/kg, Magnesium 0.14 g/kg, Selenium 0.04 g/kg, Sodium 0.14 g/kg, Zinc 4 g/kg, and vitamin E 1 IU/g. ^4^ Calculated as gross energy multiplied by metabolisability coefficient. ^5^ Calculated as dividing crude protein content by metabolisable energy content of feed. - Absent in feed type. N/A not analysed.

**Table 3:** Intake and growth performance during different phases (2–37, 38–42 and 43–57 days of age) of artificially reared lambs using three different rearing regimens: HFP57, commercial milk replacer, high fibre concentrate pellets and milk feeding to 57 days of age; HFP42, commercial milk replacer, high fibre concentrate pellets and early weaning at 42 days of age; LFP42, high protein milk replacer from 2–16 days of age followed by commercial milk replacer, low fibre concentrate pellets and early weaning at 42 days of age.

	Pre-Weaning Phase Lambs: 2–37 Days of Age					Milk Weaning Phase Lambs: 38–42 Days of Age					Post- Early Weaning Phase Lambs: 43–57 Days of Age				
Measurement	HFP57	HFP42	LFP42	Pooled SE	*p*-Value	HFP57	HFP42	LFP42	Pooled SE	*p*-Value	HFP57	HFP42	LFP42	Pooled SE	*p*-Value
Initial LW, kg	5.0	4.9	4.9	0.37	0.97	10.8	10.9	10.3	0.81	0.88	12.1	11.9	11.1	0.96	0.75
Final LW, kg	10.8	10.9	10.3	0.81	0.88	12.1	11.9	11.1	0.96	0.75	17.1	14.7	14.3	1.14	0.20
ADG, g/d	165.7	171.3	156.0	16.33	0.81	257.8	193.0	147.4	38.99	0.17	355.8 ^b^	215.6 ^a^	228.6 ^a^	0.02	<0.001
Milk replacer DMI, g/d	200.6	196.6	193.4	10.83	0.90	281.9 ^b^	138.7 ^a^	138.1 ^a^	11.95	<0.001	340.7	0.0	0.0	-	-
Pellet DMI, g/d	55.2	60.7	43.8	14.07	0.70	108.9 ^a^	269.6 ^b^	193.8 ^ab^	36.3	0.015	192.6 ^a^	448.0 ^b^	429.8 ^b^	33.42	<0.001
Lucerne chaff DMI, g/d	-	-	-	-	-	5.4	5.8	8.2	1.84	0.54	19.8	14.5	22.7	3.62	0.28
Total DMI, g/d	249.2	250.1	230.6	20.60	0.77	396.2	424.4	340.2	46.49	0.45	553.1	462.5	452.5	40.59	0.18
MEI from milk, MJ/d	4.5	4.4	4.3	0.2	0.83	6.3 ^b^	3.1 ^a^	3.1 ^a^	0.27	<0.001	7.7	0.0	0.0	-	-
MEI from pellets, MJ/d	0.7	0.8	0.5	0.2	0.66	1.4 ^a^	3.5 ^b^	2.4 ^ab^	0.46	0.014	2.5 ^a^	5.8 ^b^	5.4 ^b^	0.4	<0.001
MEI from lucerne chaff, MJ/d	-	-	-	-	-	0.04	0.05	0.07	0.01	0.54	0.2	0.1	0.2	0.03	0.14
MEI, MJ/d	5.2	5.2	4.8	0.4	0.73	7.8	6.6	5.6	0.67	0.10	10.3 ^b^	5.9 ^a^	5.5 ^a^	0.57	<0.001
CPI from milk, g/d	54.4	53.3	56.1	2.96	0.80	76.4 ^b^	37.6 ^a^	37.4 ^a^	3.05	<0.001	92.4	0.0	0.0	-	-
CPI, g/d	66.0	66.1	65.0	5.0	0.99	100	95.2	77.7	10.0	0.30	135.5 ^b^	96.3 ^a^	89.8 ^a^	8.7	0.003
Combined CP:ME ratio, g/MJ	12.6 ^a^	12.6 ^a^	13.4 ^b^	0.12	<0.001	12.8	14.2	13.8	0.18	<0.001	13.1 ^b^	16.4 ^b^	16.2 ^b^	0.05	<0.001
NDFI, g/d	13.1	14.3	5.7	3.22	0.17	29.1 ^a^	69.4 ^b^	30.5 ^a^	8.86	0.005	57.9 ^a^	115.2 ^b^	70.5 ^a^	9.0	<0.001
ADFI, g/d	4.3	4.8	2.2	1.07	0.23	11.2 ^a^	24.8 ^b^	13.7 ^a^	3.26	0.015	24.9 ^a^	42.4 ^b^	32.6 ^b^	3.8	0.01
Energy digestibility coefficient	-	-	-	-	-	-	-	-	-	-	0.93 ^b^	0.78 ^a^	0.81 ^a^	0.02	<0.001
Feed efficiency ^1^	0.65	0.66	0.66	0.02	0.93	0.65	0.41	0.41	0.07	0.055	0.65 ^b^	0.47 ^a^	0.50 ^a^	0.03	<0.001

ADFI, Acid detergent fibre intake; ADG, average daily live weight gain; CPI, crude protein intake; DMI, dry matter intake; LW, live weight; MEI, metabolisable energy intake; NDFI, neutral detergent fibre intake; SE, standard error. ^a,b^ Means in the same row with no superscript letters after them or with a common superscript letter following them are not significantly different (LSD, *p* < 0.05). ^1^ Feed efficiency calculated as LW gain/DMI.

**Table 4:** Overall intake and growth performance of lambs reared artificially (2–57 days of age) using three different rearing regimens: HFP57, commercial milk replacer, high fibre concentrate pellets and milk feeding to 57 days of age; HFP42, commercial milk replacer, high fibre concentrate pellets and early weaning at 42 days of age; LFP42, high protein milk replacer from 2–16 days of age followed by commercial milk replacer, low fibre concentrate pellets, and early weaning at 42 days of age.

	Whole Trial Period; Lambs 2 to 57 Days of Age				
Measurement	HFP57	HFP42	LFP42	Pooled SE	*p*-Value
Initial LW, kg	5.0	4.9	4.9	0.37	0.97
Final LW, kg	17.1	14.7	14.3	1.07	0.20
ADG, g/d	220.9	184.1	171.3	16.71	0.13
Milk replacer DMI, g/d	241.8 ^b^	143.0 ^a^	136.0 ^a^	10.13	<0.001
Pellet DMI, g/d	94.4 ^a^	172.8 ^b^	155.3 ^b^	17.80	0.012
Lucerne chaff DMI, g/d	5.5	3.9	6.6	0.98	0.18
Total DMI, g/d	341.7	319.7	297.8	25.45	0.51
MEI from milk, MJ/d	5.4 ^b^	3.6 ^a^	3.0 ^a^	0.30	<0.001
MEI from pellets, MJ/d	1.2 ^a^	2.2 ^b^	1.9 ^b^	0.23	0.013
MEI from lucerne chaff, MJ/d	0.04	0.03	0.05	0.01	0.22
MEI, MJ/d	6.7 ^b^	5.9 ^ab^	5.0 ^a^	0.40	0.029
CPI from milk, g/d	65.5 ^b^	38.8 ^a^	39.2 ^a^	2.76	<0.001
CPI from pellets, g/d	19.9 ^a^	36.4 ^b^	31.4 ^b^	3.74	0.013
CPI from lucerne chaff, g/d	0.7	0.5	0.9	0.13	0.18
CPI, g/d	86.1	75.7	71.4	5.80	0.22
Combined CP:ME ratio, g/MJ	12.8	13.1	14.2	0.51	0.16
NDFI from pellet, kg	1.225 ^a^	2.186 ^b^	1.116 ^a^	0.224	0.004
NDFI from lucerne chaff, kg	0.189	0.130	0.223	0.033	0.160
NDFI, g/d	25.8 ^a^	43.4 ^b^	24.4 ^a^	4.47	0.01
ADFI from pellet, kg	0.407 ^a^	0.726 ^b^	0.424 ^a^	0.075	0.008
ADFI from lucerne chaff, kg	0.150	0.104	0.177	0.027	0.160
ADFI, g/d	10.2	15.5	11.0	1.71	0.069
Feed efficiency ^1^	0.65 ^b^	0.57 ^a^	0.57 ^a^	0.02	0.009

ADFI, Acid detergent fibre intake; ADG, average daily live weight gain; CPI, crude protein intake; DMI, dry matter intake; LW, live weight; MEI, metabolisable energy intake; NDFI, neutral detergent fibre intake; SE, standard error. ^a,b^ Means in the same row with no superscript letters after them or with a common superscript letter following them are not significantly different (LSD, *p* < 0.05). ^1^ Feed efficiency calculated as LW gain/DMI.

**Table 5:** Slaughter parameters of lambs reared artificially using three different rearing regimens: HFP57, commercial milk replacer, high fibre concentrate pellets and milk feeding to 57 days of age; HFP42, commercial milk replacer, high fibre concentrate pellets and early weaning at 42 days of age; LFP42, high protein milk replacer from 2–16 days of age followed by commercial milk replacer, low fibre concentrate pellets and early weaning at 42 days of age.

Measurement	Treatment			Pooled SE	*p*-Value
	HFP57	HFP42	LFP42		
Crown to rump length, cm	74.5	70.6	71.5	1.9	0.32
Girth, cm	66.0	61.1	62.7	1.96	0.20
Ribs length, cm ^1^	27.1	27.2	26.1	0.89	0.63
Carcass weight, kg	8.0 ^b^	6.4 ^a^	6.3 ^a^	0.53	0.051
Dressing, % ^2^	470.9 ^b^	431.1 ^a^	440.8 ^a^	6.43	<0.001
Head, feet and tail, kg	1.8	1.7	1.6	0.11	0.38
Whole skin, kg	1.9	1.6	1.6	0.13	0.43
Total viscera weight, kg	4.2	4.0	3.8	0.32	0.64
Liver, g	316.7	287.2	291.7	24.67	0.66
Kidneys, g	70.3	74.3	56.1	7.34	0.22
Full stomach, kg	1.4	1.6	1.5	0.15	0.82
Full small intestine, g	960.3 ^b^	646.9 ^a^	706.3 ^a^	73.91	0.015
Empty small intestine, g	776.4 ^b^	543.1 ^a^	605.4 ^a^	54.36	0.016
Full large intestine, g	413.7	516.8	429.2	42.16	0.18
Empty large intestine, g	221.6	237.0	221.1	19.17	0.79
Blood, g	602.4	579.0	540.5	0.08	0.87
Heart, g	100.1	85.9	82.5	5.77	0.10
Lung, g	229.7	220.9	201.8	18.73	0.59
Testis, g	27.7	21.3	21.1	2.76	0.18
Spleen, g	29.4	25.0	22.8	2.04	0.10
Gutfill, kg	1.5	1.5	1.4	0.16	0.92
Omental fat, g	69.0 ^b^	35.4 ^a^	30.7 ^a^	7.47	0.003

^1^ Rib cage width along spine. ^2^ Carcass weight as a percentage of final live weight.SE, standard error. ^a,b^ Means in the same row with no superscript letters after them or with a common superscript letter following them are not significantly different (LSD, *p* < 0.05).

**Table 6:** Stomach parameters of lambs reared artificially using three different rearing regimens: HFP57, commercial milk replacer, high fibre concentrate pellets, and milk feeding to 57 days of age; HFP42, commercial milk replacer, high fibre concentrate pellets and early weaning at 42 days of age; LFP42, high protein milk replacer from 2–16 days of age followed by commercial milk replacer, low fibre concentrate pellets and early weaning at 42 days of age.

Measurement	Treatment			Pooled SE	*p*-Value
	HFP57	HFP42	LFP42		
Stomach weight					
Full stomach, kg ^1^	1.2	1.6	1.5	0.14	0.82
Empty stomach, g ^1^	338.4	421.6	381.0	27.73	0.12
Full rumen, g	972.2	1155.0	1165.8	110.89	0.40
Empty rumen, g	189.8 ^a^	274.2 ^b^	248.5 ^b^	20.13	0.019
Full reticulum, g	52.9	63.6	69.6	6.11	0.18
Empty reticulum, g	32.4	37.7	37.1	2.38	0.24
Full omasum, g	21.7 ^a^	33.6 ^b^	25.8 ^ab^	3.27	0.043
Empty omasum, g	18.4	23.0	21.4	1.81	0.20
Full abomasum, g	378.6	310.6	245.3	50.10	0.21
Empty abomasum, g	97.8	86.6	73.9	6.34	0.057
Stomach component weights relative to live weight, g/kg					
Rumen	11.0 ^a^	19.1 ^b^	17.4 ^b^	0.89	<0.001
Reticulum	2.0 ^a^	2.6 ^b^	2.6 ^b^	0.09	<0.001
Omasum	1.1 ^a^	1.6 ^b^	1.5 ^b^	0.10	0.003
Abomasum	5.8 ^b^	5.9 ^b^	5.2 ^a^	0.20	0.036
Stomach component weights relative to empty stomach weight, g/kg					
Rumen	554.9 ^a^	648.9 ^b^	651.6 ^b^	14.56	<0.001
Reticulum	96.5	90.7	98.5	4.36	0.42
Omasum	55.4	55.0	55.2	3.30	0.99
Abomasum	293.1 ^b^	205.4 ^a^	194.7 ^a^	10.54	<0.001
Body volume, L ^2^	9.5	8.3	8.3	0.68	0.36
Stomach volume					
Rumen, L ^3^	1.01	1.16	1.27	0.124	0.37
Reticulum, L ^4^	0.05	0.08	0.08	0.010	0.077
Omasum, L ^5^	0.03	0.03	0.03	0.003	0.22
Abomasum, L ^6^	1.33	1.09	0.67	0.18	0.063
Total stomach, L ^7^	2.42	2.36	2.06	0.28	0.64

^1^ Includes rumen, reticulum, omasum, and abomasum. ^2^ Calculated as body volume = $\pi \;*\left(\frac{Girth}{2\pi }\right)*\left(\frac{Girth}{2\pi }\right)*\mathrm{ribs}\;\mathrm{width}\;\mathrm{along}\;\mathrm{spine}$. ^3^ Calculated as $\mathrm{Rumen}\;\mathrm{volume}\;=\left[\frac{B}{4}*\frac{C}{2}*\frac{\mathrm{rumen}\;\mathrm{height}}{2}*\frac{4}{3}*\pi \right]+\left[\frac{B}{4}*\frac{D}{2}*\frac{\mathrm{rumen}\;\mathrm{height}}{2}*\frac{4}{3}*\pi \right]$, where B; length of rumen, C; width of dorsal rumen, D; width of ventral rumen. ^4^ Calculated as $\mathrm{Reticulum}\;\mathrm{volume}=\left[\frac{G}{2}*\frac{H}{2}*\frac{\mathrm{reticulum}\;\mathrm{height}}{2}*\frac{4}{3}*\pi \right]$, where G; length of reticulum, H; width of reticulum. ^5^ Calculated as $\mathrm{Omasum}\;\mathrm{volume}\;=\;\pi \;*{\left[\frac{J}{\pi \;*\;2}\right]}^{2}*\;I$, where I; length of omasum, J; circumference of omasum. ^6^ Calculated as $\mathrm{Abomasum}\;\mathrm{volume}\;=\left[{\left(\frac{L}{2}\right)}^{2}+\left(\frac{L}{2}*\frac{M}{2}\right)+{\left(\frac{M}{2}\right)}^{2}\right]\;*\;\pi \;*\;\frac{K}{3}$ where, K; length of abomasum, L; width at proximal section of abomasum, M; width at distal section of abomasum. ^7^ Calculated as summation of volumes of rumen, reticulum, omasum, and abomasum. SE, standard error. ^a,b^ Means in the same row with no superscript letters after them or with a common superscript letter following them are not significantly different (LSD, *p* < 0.05).

**Table 7:** Chemical composition of carcass, viscera plus blood and the whole body of lambs reared artificially using three different rearing regimens: HFP57, commercial milk replacer, high fibre concentrate pellets and milk feeding to 57 days of age; HFP42, commercial milk replacer, high fibre concentrate pellets and early weaning at 42 days of age; LFP42, high protein milk replacer from 2–16 days of age followed by commercial milk replacer, low fibre concentrate pellets and early weaning at 42 days of age.

Measurement	Baseline (Mean ± SD) *	Treatment			Pooled SE	*p*-Value
		HFP57	HFP42	LFP42		
Carcass						
Dry matter, g/kg	269.1 ± 35.0	324.2	304.3	303.8	6.2	0.51
Protein, g/kg as is	169.8 ± 7.9	182.2 ^a^	190.1 ^b^	185.5 ^a^	2.3	0.016
Fat, g/kg as is	28.5 ± 14.2	97.6 ^b^	66.4 ^a^	66.3 ^a^	6.3	0.002
Ash, g/kg as is	57.0 ± 24.3	40.5	44.8	45.7	1.9	0.060
Water, g/kg as is	730.9 ± 35.0	683.5	691.1	694.4	6.2	0.51
Gross energy, MJ/kg	-	7.84	7.23	7.24	0.2	0.16
Dry matter, kg	0.79 ± 0.11	2.18	2.14	2.11	0.05	0.51
Protein, kg	0.50 ± 0.04	1.26 ^a^	1.32 ^b^	1.26 ^a^	0.01	0.007
Fat, kg	0.08 ± 0.04	0.80 ^b^	0.44 ^a^	0.42 ^a^	0.08	0.005
Ash, kg	0.17 ± 0.07	0.28	0.30	0.32	0.01	0.13
Water, kg	2.15 ± 0.25	4.72	4.75	4.79	0.05	0.51
Gross energy, MJ	-	53.93	50.63	49.95	1.99	0.14
Viscera plus blood						
Dry matter, g/kg	227.8 ± 29.2	208.0 ^b^	191.5 ^a^	191.7 ^a^	4.7	0.030
Protein, g/kg as is	156.7 ± 12.7	136.5	139.4	138.5	1.7	0.45
Fat, g/kg as is	25.8 ± 3.7	57.2 ^b^	35.2 ^a^	37.1 ^a^	3.6	<0.001
Ash, g/kg as is	12.4 ± 3.6	9.8	10.0	10.1	0.2	0.55
Water, g/kg as is	772.2 ± 29.2	792.0	808.5	808.3	4.7	0.030
Gross energy, MJ/kg as is	-	5.62 ^b^	4.94 ^a^	4.90 ^a^	0.2	0.009
Dry matter, kg	0.39 ± 0.05	0.65	0.61	0.60	0.016	0.11
Protein, kg	0.27 ± 0.04	0.43	0.44	0.44	0.006	0.37
Fat, kg	0.04 ± 0.01	0.18 ^b^	0.12 ^a^	0.12 ^a^	0.012	0.002
Ash, kg	0.02 ± 0.01	0.03	0.03	0.03	0.001	0.59
Water, kg	1.32 ± 0.12	2.47	2.52	2.52	0.016	0.10
Gross energy, MJ	-	17.72 ^b^	15.76 ^a^	15.52 ^a^	0.553	0.034
Carcass and viscera plus blood						
Dry matter, kg	1.18 ± 0.16	2.9	2.7	2.7	0.08	0.28
Protein, kg	0.77 ± 0.08	1.7 ^a^	1.8 ^b^	1.7 ^a^	0.01	0.003
Fat, kg	0.13 ± 0.04	0.8 ^b^	0.6 ^a^	0.6^a^	0.06	0.018
Ash, kg	0.19 ± 0.06	0.3	0.3	0.3	0.01	0.16
Water, kg	3.47 ± 0.27	7.2	7.3	7.3	0.08	0.28
Gross energy, MJ	-	72.5	66.1	65.3	2.73	0.08

^a,b^ Means in the same row with no superscript letters after them or with a common superscript letter following them are not significantly different (LSD, *p* < 0.05). * Adapted from Herath et al., 2020. SD, standard deviation. SE, standard error.

**Table 8:** Dry matter, protein, fat, ash and water deposition rates in the carcass, viscera plus blood and the whole body of lambs reared artificially using three different rearing regimens: HFP57, commercial milk replacer, high fibre concentrate pellets and milk feeding to 57 days of age; HFP42, commercial milk replacer, high fibre concentrate pellets and early weaning at 42 days of age; LFP42, high protein milk replacer from 2–16 days of age followed by commercial milk replacer, low fibre concentrate pellets and early weaning at 42 days of age.

Measurement	Treatment			Pooled SE	*p*-Value
	HFP57	HFP42	LFP42		
Carcass					
Dry matter, g/d	29.6	28.3	26.7	1.34	0.25
Protein, g/d	16.7	17.3	15.5	0.05	0.18
Fat, g/d	13.3 ^b^	7.1 ^a^	6.4 ^a^	0.001	0.004
Ash, g/d	3.0	3.3	3.4	0.25	0.55
Water, g/d	58.9	56.9	55.5	1.73	0.39
Viscera plus blood					
Dry matter, g/d	6.8	5.9	5.6	0.45	0.22
Protein, g/d	4.3	4.4	4.2	0.29	0.71
Fat, g/d	2.7 ^b^	1.6 ^a^	1.5 ^a^	0. 22	0.002
Ash, g/d	0.29	0.30	0.28	0.03	0.85
Water, g/d	27.6	28.7	27.3	1.25	0.67
Carcass and viscera plus blood					
Dry matter, g/d	37.1	34.0	32.1	1.88	0.17
Protein, g/d	24.4	25.4	23.4	0.63	0.13
Fat, g/d	13.1 ^b^	9.7 ^a^	8.8 ^a^	0.11	0.017
Ash, g/d	3.4	3.5	3.6	0.25	0.58
Water, g/d	86.7	85.6	82.8	3.05	0.60

^a,b^ Means in the same row with no superscript letters after them or with a common superscript letter following them are not significantly different (LSD, *p* < 0.05). SE, standard error.

**Table 9:** Feed cost analysis of lambs reared artificially using three different rearing regimens: HFP57, commercial milk replacer, high fibre concentrate pellets and milk feeding to 57 days of age; HFP42, commercial milk replacer, high fibre concentrate pellets and early weaning at 42 days of age; LFP42, high protein milk replacer from 2–16 days of age followed by commercial milk replacer, low fibre concentrate pellets and early weaning at 42 days of age.

Cost Item (NZ$)	Treatment			Pooled SE	*p*-Value
	HFP57	HFP42	LFP42		
Milk cost/lamb	75.1 ^b^	43.1 ^a^	46.66 ^a^	3.09	<0.001
Pellet cost/lamb	4.41 ^a^	7.86 ^b^	8.12 ^b^	0.85	0.009
Lucerne chaff cost/lamb	0.72	0.52	0.82	0.13	0.260
Feed cost/lamb	80.3 ^b^	51.4 ^a^	55.60 ^a^	3.69	<0.001
Feed cost/kg live weight gain	6.76 ^b^	5.51 ^a^	5.99 ^ab^	0.32	0.018

^a,b^ Means in the same row with no superscript letters after them or with a common superscript letter following them are not significantly different (LSD, *p* < 0.05).

## Data Availability

The data presented in this study are included within the article.
